# Collagen-Derived Dipeptides and Amino Acids Have Immunomodulatory Effects in M1-Differentiated RAW264.7 Cells and PBMC

**DOI:** 10.3390/ijms24086925

**Published:** 2023-04-08

**Authors:** Takaki Tominaga, Jiapeng Huang, Shuo Wang, Miwa Noguchi, Yishan Tong, Momoko Asano-Oritani, Katsuhiko Suzuki

**Affiliations:** 1 Graduate School of Sport Sciences, Waseda University, Tokorozawa 359-1192, Japan; takaki.k-bbc@akane.waseda.jp (T.T.); hjpshidsg1234@toki.waseda.jp (J.H.); wang_sh@akane.waseda.jp (S.W.); tongyishan130@ruri.waseda.jp (Y.T.); 2 Research Fellow of Japan Society for the Promotion of Sciences, Tokyo 102-0083, Japan; 3 Unitec Foods Co., Ltd., Tokyo 103-0001, Japan; 4 Faculty of Sport Sciences, Waseda University, Tokorozawa 359-1192, Japan

**Keywords:** collagen, macrophages, cytokines, dipeptides, amino acids, glycine, proline, hydroxyproline

## Abstract

A number of food components, such as polyphenols and phytonutrients, have immunomodulatory effects. Collagen has various bioactivities, such as antioxidative effects, the promotion of wound healing, and relieving symptoms of bone/joint disease. Collagen is digested into dipeptides and amino acids in the gastrointestinal tract and subsequently absorbed. However, the difference in immunomodulatory effects between collagen-derived dipeptides and amino acids is unknown. To investigate such differences, we incubated M1 macrophages or peripheral blood mononuclear cells (PBMC) with collagen-derived dipeptides (hydroxyproline-glycine (Hyp-Gly) and proline-hydroxyproline (Pro-Hyp)) and amino acids (proline (Pro), hydroxyproline (Hyp), and glycine (Gly)). We first investigated the dose dependency of Hyp-Gly on cytokine secretion. Hyp-Gly modulates cytokine secretion from M1 macrophages at 100 µM, but not at 10 µM and 1 µM. We then compared immunomodulatory effects between dipeptides and mixtures of amino acids on M1 macrophages and PBMC. There was, however, no difference in cytokine secretion between dipeptides and their respective amino acids. We conclude that collagen-derived dipeptides and amino acids have immunomodulatory effects on M1-differentiated RAW264.7 cells and PBMC and that there is no difference in the immunomodulatory effects between dipeptides and amino acids.

## 1. Introduction

Leukocytes play an important role in inducing inflammatory responses by secreting pro-inflammatory cytokines [1,2]. These leukocytes contribute to chronic inflammation induced by obesity, aging, and inflammatory diseases [1,2,3,4]. Therefore, the regulation of leukocytes’ functions is important for human health. Recently, a number of food components, such as polyphenols and phytonutrients, have been shown to have immunomodulatory effects on leukocytes, such as macrophages [5,6,7]. Therefore, it is important to discover novel functional foods and nutrients that have immunomodulatory effects.

Collagen is the main structural protein in the skin, bones, cartilage, tendon, and blood vessels of animals, accounting for approximately 30% of the total protein content [8]. Collagen is used extensively as a material for cosmetics, pharmaceuticals, tissue engineering, and biomedical purposes [9]. Collagen is also used as a functional food and has various bioactivities, such as antioxidative effects, promoting wound healing, and relieving symptoms of bone/joint diseases [9]. Collagen is digested into dipeptides and amino acids in the gastrointestinal tract and subsequently absorbed. Therefore, various types of collagen-derived dipeptides (e.g., hydroxyproline-glycine (Hyp-Gly) and proline-hydroxyproline (Pro-Hyp)) are present in human blood after the ingestion of collagen peptide (degradation products of collagen) and meat that is rich in collagen [10,11,12,13]. Many studies have reported that collagen peptides and collagen-derived dipeptides promote wound healing [10] and muscle growth [14,15,16] as well as suppressing allergic response [17] and inflammation of endothelial cells [18]. Collagen is constituted of amino acids, such as proline (Pro), hydroxyproline (Hyp), and glycine (Gly) [8]. Gly and Hyp in particular have anti-inflammatory effects [19,20]. Furthermore, Kitakaze, et al. have reported that Hyp-Gly promotes C2C12 myoblast differentiation and myotube hypertrophy compared to a mixture of Hyp and Gly (Hyp + Gly) [14]. Therefore, collagen-derived dipeptides may have higher bioactivities compared to their respective amino acids. However, it remains unknown whether collagen-derived dipeptides and their respective amino acids have different immunomodulatory effects. In the present study, we investigated (1) the immunomodulatory effects of collagen-derived dipeptides and (2) the difference between the immunomodulatory effects of dipeptides and their respective amino acids using murine macrophages and human peripheral blood mononuclear cells (PBMC) including lymphocytes.

## 2. Results

### 2.1. Inflammatory Cytokine Secretion by M1 Macrophages

M1 macrophages are representative macrophages that secrete inflammatory cytokines. Therefore, we first investigated whether M1 macrophages exhibited enhanced cytokine secretion compared to unstimulated M0 macrophages using murine macrophage cell line RAW264.7 cells. In this study’s protocol, M1 macrophages exhibited enhanced interleukin (IL)-1β, IL-6, and tumor necrosis factor-α (TNF-α) secretion (Table 1).

### 2.2. Hyp-Gly Inhibits IL-1β Secretion by M1 Macrophages

**Figure 1:**
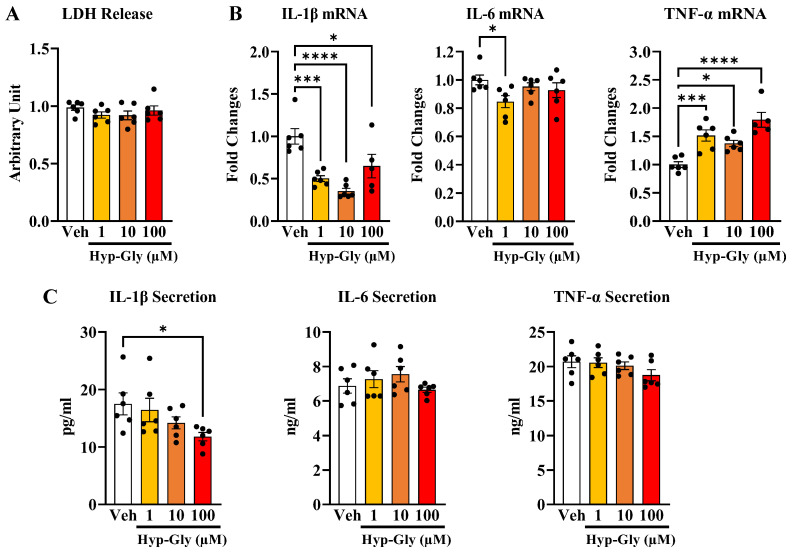
The concentration-dependent effect of Hyp-Gly on M1 macrophage (**A**) LDH release (*n* = 6), (**B**) inflammatory cytokine gene expression (*n* = 5–6), and (**C**) inflammatory cytokine secretion (*n* = 6). Data are shown as means ± SE. Data were analyzed by one-way ANOVA followed by Dunnett’s post hoc test compared to vehicle (Veh) [* *p* < 0.05, *** *p* < 0.001, **** *p* < 0.0001].

Next, we investigated the dose dependence of the immunomodulatory effects of Hyp-Gly on M1 macrophages. We first assessed the effects of Hyp-Gly in terms of M1 macrophage cellular damage. Hyp-Gly did not influence the release of the cellular damage marker lactate dehydrogenase (LDH) (Figure 1A). We then investigated the effects of Hyp-Gly on inflammatory cytokine gene expression. Hyp-Gly inhibited IL-1β and IL-6 gene expression and increased TNF-α gene expression (Figure 1B). We next investigated the effects of Hyp-Gly on inflammatory cytokine secretion. Hyp-Gly inhibited IL-1β secretion at 100 µM (Figure 1B). However, Hyp-Gly did not change IL-6 and TNF-α secretion (Figure 1C). These results indicate that Hyp-Gly has immunomodulatory effects at 100 µM without causing cellular damage on murine M1 macrophages.

### 2.3. Comparison of the Immunomodulatory Effects of Dipeptides and Amino Acids on M1 Macrophages

**Figure 2:**
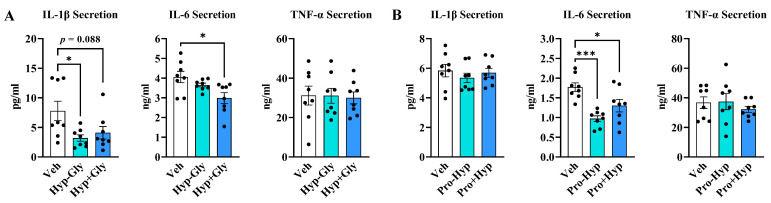
(**A**,**B**) The comparison of the effects of dipeptides and amino acids (each at 100 µM) on inflammatory cytokine secretion by M1 macrophages (*n* = 8). Data are shown as means ± SE. Data were analyzed by one-way ANOVA followed by Tukey’s post hoc test [* *p* < 0.05, *** *p* < 0.001]. Hyp-Gly and Pro-Hyp refer to dipeptides. Hyp + Gly means a mixture of Hyp and Gly. Pro + Hyp means a mixture of Pro and Hyp.

We then compared the immunomodulatory effects of Hyp-Gly versus Hyp + Gly on M1 macrophages. Hyp-Gly inhibited IL-1β secretion, and Hyp + Gly tended to inhibit IL-1β secretion (Figure 2A). Hyp + Gly inhibited IL-6 secretion, but not Hyp-Gly (Figure 2A). However, there was no difference in the inhibitory effects between Hyp-Gly and Hyp + Gly on IL-1β and IL-6 secretion (Figure 2A). Hyp-Gly and Hyp + Gly did not change TNF-α secretion (Figure 2A). Pro-Hyp is another collagen-derived dipeptide [10]. We next investigated the immunomodulatory effects of Pro-Hyp and a mixture of Pro and Hyp (Pro + Hyp). Both Pro-Hyp and Pro + Hyp inhibited IL-6 secretion (Figure 2B). However, there was no difference in the inhibitory effects between Pro-Hyp and Pro + Hyp on IL-6 secretion (Figure 2B). Pro-Hyp and Pro + Hyp did not change IL-1β and TNF-α secretion (Figure 2B). These results indicate that although dipeptides also have immunomodulatory effects on murine M1 macrophages, there are no differences in the immunomodulatory effects between dipeptides and amino acids.

### 2.4. Comparison of the Immunomodulatory Effects of Dipeptides and Amino Acids on PBMC

**Figure 3:**
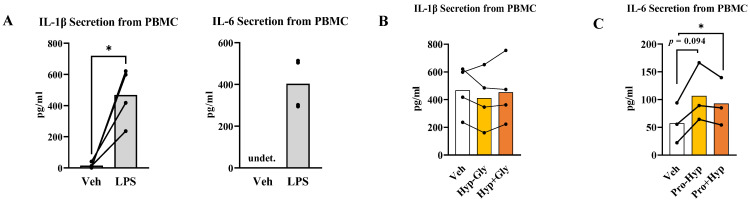
(**A**) Inflammatory cytokine secretion from LPS-stimulated PBMC (*n* = 4). (**B**,**C**) The comparison of the effects of dipeptides and amino acids (each at 100 µM) on inflammatory cytokine secretion by PBMC (*n* = 3–4). The lined plots are the data from the same subjects. Data are shown as means. Data were analyzed by (**A**) repeated *t*-test or (**B**,**C**) repeated one-way ANOVA followed by Tukey’s post-hoc test [* *p* < 0.05]. Hyp-Gly and Pro-Hyp refer to dipeptides. Hyp + Gly means a mixture of Hyp and Gly. Pro + Hyp means a mixture of Pro and Hyp.

We next investigated that collagen-derived dipeptides also have similar bioactivities on human-derived leukocytes. Then, we compared the immunomodulatory effects of dipeptides and amino acids using lipopolysaccharide (LPS)-stimulated human PBMC. At first, we investigated whether LPS-stimulated PBMC exhibited enhanced cytokine secretion. In this study’s protocol, LPS stimulation promoted IL-1β and IL-6 secretion (Figure 3A). Then, we compared the immunomodulatory effects of dipeptides and amino acids. Both Hyp-Gly and Hyp + Gly did not change IL-1β secretion (Figure 3B). Pro-Hyp tended to increase IL-6 secretion, and Pro + Hyp increased IL-6 secretion (Figure 3C). However, there was no difference in the increasing effects between Pro-Hyp and Pro + Hyp on IL-6 secretion (Figure 3C). These results indicate that although dipeptides also have immunomodulatory effects on human PBMC, there are no differences in the immunomodulatory effects between dipeptides and amino acids.

## 3. Discussion

The purpose of this study is to investigate (1) whether collagen-derived dipeptides have immunomodulatory effects and (2) whether there is a difference between the immunomodulatory effects of dipeptides and their respective amino acids. Our findings indicate that both collagen-derived dipeptides and amino acids have immunomodulatory effects on murine M1 macrophages and human PBMC, but there is no difference in the bioactivities between dipeptides and their respective amino acids. Specifically, we observed that Hyp-Gly and Pro-Hyp inhibited IL-1β and IL-6 secretion in M1-differentiated RAW264.7 cells, respectively. These findings are consistent with those of Kouguchi, et al., who have reported that Hyp-Gly and Pro-Hyp inhibited TNF-α-induced IL-8 and soluble intercellular adhesion molecule-1 (sICAM-1) secretion in human umbilical vein endothelial cells (HUVECs) [18]. Some previous studies show that dipeptides have higher bioactivities than amino acids [14,17]. For example, Nishikami, et al. have reported that Pro-Hyp promotes antigen-induced regulatory T cell development but not Pro + Hyp [17]. Kitakaze, et al. have reported that Hyp-Gly promotes C2C12 myoblast differentiation and myotube hypertrophy rather than Hyp + Gly [14]. Contrary to these studies, we did not observe any significant differences in bioactivity between dipeptides and their respective amino acids in M1-differentiated RAW264.7 cells or LPS-stimulated PBMC. Although some studies show higher dipeptides bioactivities [14,17], the mechanisms behind this remain unclear. In the present study, Hyp-Gly inhibited IL-1β secretion but not Pro-Hyp in M1 macrophages, which suggests that Gly regulates IL-1β secretion. In view of the fact that Gly has anti-inflammatory effects [19], Gly may also exert immunomodulatory effects in the present study. It is known that collagen-derived dipeptide concentrations transiently increase in human blood after 10–25 g of collagen peptide intake, eventually reaching 10–100 µM [11,12,13]. In the present study, we observed the immunomodulatory effects of dipeptide at 100 µM in vitro. Therefore, dietary collagen peptide intake may also have immunomodulatory effects on leukocytes.

In contrast to the findings in M1 macrophages, the present study observed that dipeptides promoted IL-6 secretion in LPS-stimulated PBMC. PBMC is composed of various cell types, such as lymphocytes, monocytes, and natural killer cells [21]. Among them, lymphocytes, particularly T cells, are a predominant population [21]. Our study also revealed that more than 80% of the isolated PBMC were lymphocytes (see Section 4.2). Therefore, it is possible that dipeptides induce distinct responses in M1 macrophages and lymphocytes. The present study also showed that Hyp-Gly inhibited IL-1β secretion in M1 macrophages but not PBMC. Because PBMC has higher IL-1β gene expression levels than isolated monocytes and macrophages [22], the effects of Hyp-Gly may be masked by other leukocytes than monocytes.

In the present study, IL-1β and TNF-α secretion levels did not parallel gene expression levels on M1 macrophages. Although regulating transcriptional levels play a role in regulating the secretion levels of IL-1β and TNF-α, regulating release levels are also important [23]. Initially, IL-1β is produced as inactive pro-IL-1β [23]. Then, pro-IL-1β is cleaved by caspase-1 and released as mature IL-1β [23]. To activate caspase-1, a multiprotein complex termed the inflammasome is necessary [23]. Inflammasome is formed from the NOD-like receptor family, pyrin domain-containing 3 (NLRP3), NOD-like receptor C4 (NLRC4), absent in melanoma 2 (AIM2), and other proteins [23]. In the present study, Hyp-Gly may influence the inflammasome, leading to the unparallel dynamics between IL-1β gene expression and secretion levels. Similarly, TNF-α is produced as a transmembrane TNF-α (pro-TNF-α) [23]. Then, pro-TNF-α is cleaved by enzymes, such as TNF-α converting enzyme (TACE), and released as soluble TNF-α. In the present study, Pro-Hyp may influence TNF-α transcription but not TACE, resulting in a difference between TNF-α gene expression and secretion levels. 

LPS stimulates M1 macrophage polarization and proinflammatory cytokine secretion via the TLR4-nuclear-factor kappa-light-chain-enhancer of the activated B cells (NF-kB) pathway [24]. This study showed that LPS/IFN-γ stimulation promoted IL-1β, IL-6, and TNF-α secretion. However, Hyp-Gly only inhibited IL-1β secretion, and Pro-Hyp only inhibited IL-6 secretion. Ji, et al. have reported that dietary Hyp supplementation attenuates dextran-sulfate-sodium-induced colonic inflammation through NF-kB in mice, and Hyp attenuates LPS-induced IL-6 gene expression through NF-kB in RAW264.7 macrophages [20]. Therefore, the immunomodulatory effects of Hyp-Gly and Pro-Hyp may be mediated by NF-kB attenuation. However, since Hyp-Gly and Pro-Hyp only inhibited the secretion of specific cytokines, there may be other mechanisms besides NF-kB.

Peptide histidine transporter 2 (PHT2, also known as SLC15A3), one of the peptide transporters, is highly expressed on macrophages and increased by LPS stimulation in a manner dependent on NF-kB [25]. Therefore, the immunomodulatory effects of collagen-derived dipeptides may be mediated by PHT2. In the present study, we found that different dipeptides inhibit different cytokines. Therefore, Hyp-Gly and Pro-Hyp may have other mechanisms. One possible mechanism for the immunomodulatory effects of dipeptides is the modulation of the cellular metabolism of immune cells (immunometabolism). Recently, it has been shown that cellular metabolism (e.g., amino acid metabolism) is important in regulating macrophage function and cytokine secretion [26]. Gly is a substrate of glutathione, which is an antioxidant that protects from reactive oxygen species, restricting the activation of NF-kB via glutathione metabolism [26]. Although the detailed mechanisms are unknown, Hyp inhibits the LPS-induced NF-kB activation of macrophages [20]. Therefore, supplementation of Hyp-Gly and Pro-Hyp may change the cellular metabolism and redox balance, resulting in the inhibition of cytokine secretion. Further studies are necessary to investigate the downstream pathway after dipeptide uptake into the cells.

LPS is a ligand of toll-like receptor 4 (TLR4). Because fatty acids are also ligands of TLR4 [3], collagen-derived dipeptides and amino acids may reduce fatty-acids-induced inflammation. Furthermore, a previous study has shown that collagen-derived dipeptides inhibit TNF-α-induced inflammatory responses in endothelial cells [17]. Therefore, collagen-derived dipeptides may have immunomodulatory effects on other inflammatory models and other cell types. In this study, we only used RAW264.7 cells and PBMC and did not investigate other cell lines. Therefore, further experiments are necessary to investigate the immunomodulatory effects on various types of cells, including non-immune cell lines. Moreover, this study only investigated the immunomodulatory effects in the context of LPS/IFN-γ stimulation. Further studies are necessary using other macrophage stimulators, such as fatty acids, zymosan, and inflammatory cytokines.

## 4. Materials and Methods

### 4.1. Cell Culture for M1 Macrophages

The cell culture of RAW264.7 cells and M1 macrophage differentiation protocols were described previously but with minor modifications [27]. RAW264.7 murine macrophages were maintained in high-glucose Dulbecco’s modified Eagle’s medium (DMEM) with 10% fetal bovine serum (FBS) at 37 °C in a 5% CO_2_ atmosphere for cell proliferation. Then, RAW264.7 cells were incubated with differentiation medium (DM) for 4 h to differentiate M1 macrophages. DM is FBS-free DMEM with LPS (from *Escherichia coli* 055: B5, 1 μg/mL, Sigma-Aldrich, St. Louis, MO, USA) and interferon-γ (IFN-γ, 20 ng/mL, BioLegend, San Diego, CA, USA). After a 4 h differentiation period, a further incubation with the mixture of DM and collagen-derived dipeptides/amino acids was conducted for 20–24 h. After the incubation, the culture medium was centrifuged at 10,000× *g* for 5 min to remove cell debris and stored at −80 °C until further analysis. The culture protocols are detailed below.

The experiment for Result 2.1: The cells were seeded into 6-well plates (2 × 10^5^ cells/well) and incubated in DMEM with 10% FBS for cell proliferation. After incubation for 24 h, the culture medium was replaced with 1.8 mL of FBS-free DMEM (M0) or DM (M1) and incubated for a further 28 h (*n* = 6 in each group). The experiment for Result 2.2: The cells were seeded into 6-well plates (2 × 10^5^ cells/well) and incubated in DMEM with 10% FBS for cell proliferation. After incubation for 24 h, the culture medium was replaced with 1.8 mL DM. After incubation for 4 h, Hyp-Gly (Bachem, Bubendorf, Switzerland) was added to DM to final concentrations of 1, 10, and 100 µM and then incubated for a further 24 h (*n* = 6 in each group). The experiment for Result 2.3: The cells were seeded into 24-well plates (5 × 10^5^ cells/well) and incubated in DM. After incubation for 4 h, 100 µM of dipeptides (Hyp-Gly; Pro-Hyp, Bachem, Bubendorf, Switzerland) or 100 µM of individual amino acids (Gly, Sigma-Aldrich, St. Louis, MO, USA; Hyp, Nacalai Tesque, Kyoto, Japan; Pro, Sigma-Aldrich St. Louis, MO, USA; Hyp + Gly is a mixture of 100 µM of Hyp and 100 µM of Gly, Pro + Hyp is a mixture of 100 µM of Pro and 100 µM of Hyp) were added to 350 µL DM and incubated for a further 20 h. Vehicle (Veh) was used as a control (*n* = 8 in each group).

### 4.2. Cell Culture for PBMC

PBMC was isolated from the whole blood of seven healthy donors using density gradient-centrifugation. Histopaque 1119 (4 mL; Sigma-Aldrich, St. Louis, MO, USA), Histopaque 1077 (4 mL; Sigma-Aldrich, St. Louis, MO, USA), and heparinized whole blood (4 mL) was added gradually to a 15 mL tube in this order and centrifuged at 1600× *g* for 30 min. Then, the upper cellular layer was isolated and washed with Hanks’ Balanced Salt solution (HBSS) at 900× *g* for 10 min two times. The number of isolated cells was counted using an automatic blood cell counter (pocH-100i, Sysmex Co., Kobe, Japan), and more than 80% of the isolated PBMC were lymphocytes. Then, these cells were seeded into 24-well plates (7 × 10^5^ cells/well for Results 2.4, Figure 3A,B; 1 × 10^5^ cells/well for Results 2.4, Figure 3C), and HBSS with LPS (from *Escherichia coli* 055: B5, Sigma-Aldrich, St. Louis, MO, USA), dipeptides (Hyp-Gly; Pro-Hyp, Bachem, Bubendorf, Switzerland), and individual amino acids (Gly, Sigma-Aldrich, St. Louis, MO, USA; Hyp, Nacalai Tesque, Kyoto, Japan; Pro, Sigma-Aldrich St. Louis, MO, USA; Hyp + Gly is a mixture of 100 µM of Hyp and 100 µM of Gly, Pro + Hyp is a mixture of 100 µM of Pro and 100 µM of Hyp) were added to final concentrations/volume of 1 μg/mL LPS, 100 µM of dipeptides, 100 µM of individual amino acids, and 300 μL HBSS/well. After incubation for 24 h, the culture medium was centrifuged at 10,000× *g* for 5 min to remove cell debris and stored at −80 °C until further analysis.

This study protocol on human subjects is approved by The Academic Research Ethics Committee of Waseda University (2021-108). Before the research began, all subjects signed an informed consent form.

### 4.3. ELISA Analysis

The concentrations of murine IL-1β, IL-6, and TNF-α in the cell culture medium were measured by DuoSet^®^ ELISA Kit (R&D Systems, Minneapolis, MN, USA,#DY401, #DY406, and #DY410, respectively). Although the assay range of IL-1β is 15.6–1000 pg/mL, we checked that an IL-1β DuoSet^®^ ELISA kit could measure up to 1.9 pg/mL using further diluted standards. The concentrations of human IL-1β and IL-6 in the culture medium were measured by a Quantikine^®^ High Sensitivity ELISA Kit (R&D Systems, Minneapolis, MN, USA, #HSLB00D, #HS600C, respectively). The absorbance was measured using a Spectra Max iD5 device (Molecular Devices Inc., San Jose, CA, USA).

### 4.4. LDH Assay

We used a Cytotoxicity LDH Assay Kit-WST (Dojindo Molecular Technologies, Inc., Kumamoto, Japan). The absorbance was measured using a Spectra Max iD5 device (Molecular Devices Inc., San Jose, CA, USA).

### 4.5. Real-Time PCR

We performed real-time PCR as previously described [5,7,28]. The detailed protocol is as follows: Total RNA was extracted from the cultured cells using TRIzol^®^ Reagent (Invitrogen, Carlsbad, CA, USA). The purity and concentrations of the extracted total RNA were measured using a NanoDrop^®^ ND-1000 (Thermo Fisher Scientific, Waltham, MA, USA). The total RNA was reverse-transcribed into cDNA using a High-Capacity cDNA Reverse Transcription Kit (Applied Biosystems, Foster City, CA, USA). Real-time PCR was performed using a Fast 7500 Real-Time PCR system (Applied Biosystems, Foster City, CA, USA) and Fast SYBR™ Green Master Mix (Applied Biosystems, Foster City, CA, USA). The PCR conditions consisted of one denaturation cycle at 95 °C for 20 s, 40 cycles consisting of denaturation at 95 °C for 3 s, and annealing and elongation at 60 °C for 30 s. Each gene was normalized using 18s rRNA. All data were calculated using the ΔΔCt method. The specific primer sequences were as follows (forward and reverse); 18s rRNA, 5′-TTCTGGCCAACGGTCTAGACAAC-3′ and 5′-CCAGTGGTCTTGGTGTGCTGA-3′; TNF-α, 5′-CCTCCCTCTCATCAGTTCTA-3′ and 5′-ACTTGGTGGTTTGCTACGAC-3′; IL-1β, 5′-GGGCCTCAAAGGAAAGAATC-3′ and 5′-ACTTGGTGGTTTGCTACGAC-3′; and IL-6, 5′-TAGTCCTTCCTACCCCAATTTCC-3′ and 5′-TTGGTCCTTAGCCACTCCTTC-3′.

### 4.6. Statistical Analysis

The data are expressed as means ± the standard error (SE). One-way analysis of variance (ANOVA) followed by Dunnett’s post-hoc test was used for the data of Figure 1A–C. One-way ANOVA followed by Tukey post-hoc test was used for the data of Figure 2A,B. A repeated *t*-test was used for the data of Figure 3A. A repeated one-way ANOVA followed by Tukey post-hoc test was used for the data of Figure 3B,C. The statistical analysis was performed using Prism software (v.9.2.0, GraphPad, San Diego, CA, USA) with statistical significance being defined as *p* < 0.05.

## 5. Conclusions

The purpose of this study is to investigate (1) whether collagen-derived dipeptides have immunomodulatory effects and (2) whether there is a difference between the immunomodulatory effects of dipeptides and their respective amino acids. In this study, we found that collagen-derived dipeptides and amino acids exert immunomodulatory effects on M1-differentiated RAW267.4 cells and PBMC. However, there was no difference in the immunomodulatory effects between dipeptides and amino acids. These findings provide information about the bioactivities of collagen-derived dipeptides. Further studies are necessary to investigate the bioactivities of collagen-derived dipeptides.

## Figures and Tables

**Table 1:** Inflammatory cytokine secretion by undifferentiated (M0) and M1 macrophages (*n* = 6).

	M0 (pg/mL)	M1 (pg/mL)
IL-1β	Undetectable	17.50 ± 1.89
IL-6	Undetectable	6873.23 ± 411.31
TNF-α	86.85 ± 4.30	20,693.60 ± 865.54

Data are shown as means ± SE.

## Data Availability

The raw data used in this study are available upon request from the first author.
